# A Naturalistic Intervention to Promote Executive Functions in Primary School Children: A Pilot Study

**DOI:** 10.3390/brainsci14010070

**Published:** 2024-01-10

**Authors:** Jonatas B. Souza, Bruna T. Trevisan, Liana G. Nunes, Wagner L. Machado, Alessandra G. Seabra

**Affiliations:** 1 Post-Graduation Program in Human Development Sciences, CCBS—Mackenzie Presbyterian University, Rua da Consolação, nº 930, São Paulo 01302-907, SP, Brazil; brunattrevisan@gmail.com (B.T.T.); 72006374@mackenzista.com.br (L.G.N.); alessandra.seabra@mackenzie.br (A.G.S.); 2 Graduate Program in Psychology, Pontifical Catholic University of Rio Grande do Sul, PUCRS Av. Ipiranga, 6681—Building 11—9th Floor—Room 930—Parthenon, Porto Alegre 90619-900, RS, Brazil; wagner.machado@pucrs.br

**Keywords:** cognitive intervention, executive functions, cognitive control, working memory, inhibitory control, mental flexibility, naturalistic intervention

## Abstract

Executive functions are related to the control of cognition, emotion, and behavior. They are essential to lifelong outcomes, including school performance. Naturalistic interventions embedded in children’s daily activities and environments have greater effects. Therefore, this pilot study aimed to develop a naturalistic program suitable for schools, based on Goal Management Training (GMT), and to analyze its effects on executive functions and behavior. The participants consisted of 35 students from 2nd to 5th grade with executive dysfunction complaints. They underwent neuropsychological assessments of working memory, inhibition, cognitive flexibility, and intellectual capacity. Teachers and parents answered questionnaires on executive functions and behavior. Students were randomly assigned to an active control group, who participated in sessions on citizenship, and an experimental group (EG), stimulated through the executive function program, both with 16 sessions conducted by psychologists. After the intervention, all participants were reevaluated. The two-way Wald-type statistic (WTS) revealed greater improvement in executive functions for the EG, including working memory and inhibition. Additionally, parents and teachers, blind to the experimental conditions, reported improvements in some measures of executive functions and behavior. The results are encouraging, but further studies should test the intervention when implemented with larger samples and by teachers.

## 1. Introduction

### 1.1. Naturalistic Intervention and Executive Functions

Naturalistic interventions are characterized as procedures for teaching skills and behaviors occurring within the context of natural activities [1,2]. Such interventions have shown an increased potential for creating more generalizations and spontaneity, as well as enhancing the maintenance of the target behavior compared to interventions in non-naturalistic contexts [3]. As these procedures are implemented in the environment where the skills will be required, the gains are more rapidly produced, with a greater likelihood of being generalized and sustained after the intervention [2,4].

Generally, a naturalistic intervention program tends to incorporate a parental education component, which teaches caregivers how to implement the procedures within a child’s daily routine. Effective parental involvement can positively enhance outcomes and improve the emotional relationship between both parties [5,6]. Another form of naturalistic intervention involves teachers and friends, whose involvement also contributes to enhancing gains. In a naturalistic intervention procedure, teachers can be guided to stimulate the required skills within the school context, aiming to engage children experiencing impairment in school activities and effectively include them with their peers [6]. In other words, naturalistic intervention seeks to align with the routines of individuals undergoing the intervention and can be easily generalized to their own social environment. Moreover, in the context of childhood, naturalistic intervention can extend or modify experiences that typically occur within the child’s environment [2].

Many school difficulties faced by children and adolescents have been associated with impairments in executive functions [7]. Executive functions are linked to top-down mental processes necessary for the ability to resist environmental distractions, plan actions, set goals, inhibit automatic behaviors, flexibly solve problems, and analyze consequences [7,8]. These skills encompass cognitive flexibility, working memory, and inhibitory control. Cognitive flexibility involves the skill of thinking about something in different ways. Working memory concerns to the manipulating of information in mind, for example, when a reader organizes different pieces of information into a reading comprehension. Inhibitory control is the process responsible for ignoring a distraction, stopping an impulsive utterance, or overcoming automatic behaviors [7]. Stimulating executive functions is related to better academic performance in children and adolescents and plays a crucial role in cognitive, social, and emotional development throughout childhood [7,9,10].

In children, executive function intervention can be especially important, as childhood is a period characterized by significant improvements in these functions. Although a strong increase in executive functions occurs between 4 and 6 years old, during middle childhood, particularly up to 10 years of age, there are important progressions in working memory, inhibitory control, and cognitive flexibility [11,12]. As children engage in increasingly complex tasks at school and in social environments, the demands on their executive functions increase. Moreover, interventions and experiences during this phase can play a crucial role in shaping the trajectory of executive function development, highlighting the importance of targeted educational and cognitive interventions to support optimal cognitive growth in children in the middle childhood [11].

Among different types of intervention, numerous studies have reported positive effects from adaptations in school curricula for the development of executive functions [13], such as Tools of the Mind [14], Promoting Alternative Thinking Strategies [15], Intervention Program for Self-regulation and Executive Functions [16], and the Chicago School Readiness Project [17], which are considered activities supplementary to the existing curriculum. Additionally, Montessori curriculum has been assuming an important role in promoting self-regulation, goal-directed behavior, self-monitoring, and flexibility to solve problems and revise plans [18]. Another intervention model supported by scientific evidence is Goal Management Training (GMT) [19], as discussed in the following section.

### 1.2. Goal Management Training

GMT [20] is a structured rehabilitation protocol which promotes a metacognitive intervention, based on Duncan’s theory of “goal neglect”, originally developed by Robertson [21]. Duncan, in his theoretical model, describes that a complex action should be hierarchically structured to achieve a specific goal. Individuals with executive dysfunctions often neglect these goals and consequently fail to direct their behaviors toward them. Therefore, a discrepancy between behavior and goal is observed [21]. The GMT intervention involves guiding individuals to periodically stop what they are doing, review task goals, assess their progress, and monitor or check their performance as they proceed through each step of the intended objective.

The GMT model also includes activities such as mindfulness practice and psychoeducation, employing self-instruction and metacognition techniques. Mindfulness practice has been used in school contexts and has proven effective in promoting gains in executive functions [22]. Psychoeducation, with self-instruction and metacognition, generally involves direct teaching in problem-solving, organization, self-monitoring, and self-regulation using internal speech as a mediator [23]. As a comprehensive approach, GMT not only introduces mindfulness techniques, emphasizing the idea of ‘present’ and ‘absent mind,’ but also highlights psychoeducation, in addition to the cognitive training itself. At the end of each session, participants are provided with cognitive training activities and encouraged to use the trained techniques in their daily lives [24]. Thus, the stages consist of: stop and ask “what am I doing?”; check the mental blackboard; define the main task or goal to be met; list the steps or stages required to meet a goal; learn the steps; do the steps; check if you did what you planned [24].

### 1.3. Empirical Evidence

The GMT program has demonstrated its efficacy in various contexts. For instance, Tornas et al. [24] examined the effectiveness of GMT training on executive function domains in patients with chronic acquired brain injury. They conducted a blind, randomized study in which the experimental group (*n* = 33) underwent GMT training, while the control group was exposed only to psychoeducational lectures (*n* = 37). Subjects underwent neuropsychological evaluation to measure immediate post-intervention results and follow-up at 6 months. The study followed some adaptations from the initial protocol proposed by Levine et al. [19]; however, all sessions adhered to the original procedure, including an introduction to key concepts, practical exercises, and a discussion of real-life examples. Starting from the fourth session, all participants received a text message on their cell phones with the word “STOP”, corresponding to the key instruction of the GMT procedure, to remind participants to use the learned strategies during the day. There was also an additional session that aimed, through the proposed training techniques, to teach participants to regulate their emotions. The results showed that, after the intervention, there were improvements in the experimental group in tasks requiring attentional control and subjective executive function complaints. The strongest effects were observed six months after the intervention, suggesting that the learned strategies were applied and consolidated after the training. Overall, the results demonstrated that individuals with acquired brain injury subjected to GMT training exhibited more favorable effects in executive function cognitions, particularly executive attention, compared to exposure to psychoeducation alone [24].

In a meta-analysis, Stamenova and Levine [25] sought to verify the effectiveness of GMT, either alone or in combination with other approaches, in improving executive functions in adult individuals. The measured cognitive domains included daily executive functioning tasks, working memory, processing speed, long-term memory, general mental health, daily living activities, and patients’ subjective perception of executive tasks. A total of 21 publications were analyzed. The results indicated small to moderate effect sizes across all measured cognitive domains, except for information processing speed. These effect sizes were maintained in subsequent follow-up assessments in the published studies. The results support GMT as an effective intervention in rehabilitating executive functions in adults, with effects that persist in follow-up evaluations [25].

Concerning the child and adolescent population, Nunes and Seabra [26] adapted the GMT program for adolescents aged 11 to 17 with complaints regarding executive functions in the social environment. The study included neuropsychological assessments with scales, traditional tests, and ecological activities. Initially, the participants were divided into 11–13 years of age and 14–17 years of age and then randomly allocated between experimental and control groups. The intervention resulted in gains in measures of inhibitory control, cognitive flexibility, and working memory [26]. The majority of these studies used traditional tests to assess inhibitory control, working memory, planning, flexibility, and problem resolution. Only two of these studies incorporated ecological tasks as a way to measure these domains in naturalistic setting [24,26].

In summary, these studies, using the GMT principles, with activities and examples from daily life, have shown the generalization of strategies learned in the research setting to the social environment of participants [19,25,26]. In this way, in the present study, a program was developed for primary school students, particularly those in grades 2 to 5 of elementary education. It should be highlighted that in Brazil, this schooling period is critical as it marks the transition from primary school to secondary school, a more complex period in terms of school dynamics, featuring different subjects and teachers that require more autonomy, organization, and planning from students. Therefore, teachers must provide students with activities within the school context that develop their executive functions in the same way that the academic content is developed. This will assist students in transitioning to secondary school. In this context, the present pilot study aimed to identify the gains from an executive function intervention based on the Goal Management Training approach for students in the final grades of primary school.

## 2. Materials and Methods

### 2.1. Participants

In the present study, 35 students, aged 8 to 10 years, from the second to fifth grade of a state public school in the city of São Paulo, Brazil, participated. Participants expressed complaints regarding executive functions according to reports from parents and teachers, as described in the procedure.

Of the 45 children initially recommended by the teachers, two parents did not agree to their participation in the study. Therefore, the study continued with 43 students. A series of neuropsychological measures were applied before and after the interventions to characterize the participants and assess the impact of the intervention program. Criteria for the exclusion of students included presenting a previous psychiatric condition, Intelligence Quotient (IQ) below 70, a history of neurological injury, or disorders according to the school’s or parents’ report. Two participants were excluded from this study after the teachers’ indication due to a previous psychiatric condition reported by the school, and two participants were excluded after the first assessment due to presenting an IQ below 70. Two children were absent from school for health reasons and two children changed schools, throughout the intervention phase, resulting in their withdrawal from the study, including two children from the control group and two from the experimental group. The final sample was composed of 35 children. Participants did not undergo any other psychological intervention during the study.

### 2.2. Assessment Instruments

The choice of instruments was made to include the basic components of executive functions (working memory, cognitive flexibility, and inhibition), as well as complementary behavioral measures based on the report of parents and teachers.

For evaluating functional performance regarding parental and teacher perceptions in two different environments, school, and home, the Inventory of Executive Functioning Difficulties, Regulation, and Delay Aversion for Children (Inventário de Dificuldades em Funções Executivas, Regulação e Aversão ao Adiamento para crianças—IFERA-I) was used [27]. The inventory was designed to be an instrument encompassing the complexity of Attention Deficit Hyperactivity Disorder (ADHD) neuropsychology, including sub-scales of executive functions (inhibition, working memory, and flexibility), delay aversion, and regulation. However, it also proved useful for evaluating non-clinical samples, showing good predictive roles for behavior indices and with robust psychometric properties across a wide age range, from 3 to 14 years of age [27,28]. The instrument consists of 28 items, such as “When they want something, they expect it immediately” and “Has difficulty waiting when they know they will receive a gift.” Each response is given on a five-point Likert-type scale (never, rarely, sometimes, often, always). Higher scores indicate more difficulties. The items are randomly distributed but represent 5 subscales: working memory; inhibitory control; cognitive flexibility; delay aversion; and self-regulation. It can be answered by parents and teachers. In this study, scores for each scale and the instrument’s total were used. IFERA-I has validity evidences for children from four to fourteen years old, including content validity, according to judge analysis and to confirmatory factor analysis, and correlation with other executive functions instruments, as well as test–retest reliability [27].

To assess behavior and mental health problems in children, the Strengths and Difficulties Questionnaire (SDQ) was employed [29]. Versions for both parents and teachers were used. The SDQ comprises twenty-five items, divided into five subscales: Emotional problems, Conduct problems, Hyperactivity, Peer relationship problems, and Prosocial behavior. Scores range from 0 to 10 within each subscale, and the total score varies between 0 and 40. Higher scores indicate more difficulties, except for in Prosocial behavior. The SDQ was validated for the Brazilian population by Woerner et al. [30].

To evaluate the IQ, the Portuguese version of the Wechsler Abbreviated Scale of Intelligence (WASI) was utilized [31]. This is an individually administered instrument applicable to participants aged between 6 and 89 years. The battery comprises four subtests: Vocabulary, Cubes, Similarities, and Matrices Reasoning. In this study, *T*-scores for total IQ were used, according to the Brazilian norms [32].

For assessing working memory, the Digits subtest of the WISC-IV (Wechsler, 2013) was applied [33]. The subtest has two parts, forward order, and reverse order. Initially, the forward order was administered, where the examinee was required to repeat sequences of numbers spoken by the administrator. Then, in the backward order, the examinee had to repeat sequences of numbers in reverse. Weighted scores in the Digits subtest were used.

To assess inhibitory control, the Go/No-Go Test was utilized [34]. The test is computerized and presents red or blue squares. The child’s task is to respond by pressing a button on the touchscreen computer monitor, only when the stimulus is red (Go items), inhibiting the action for blue items (No-Go items). The instrument comprises four parts with 40 items each. In the first part, target stimuli account for 25% of the items presented at a speed of 2000 milliseconds. In the second part, target stimuli account for 75% of the items, presented at a speed of 1000 milliseconds. In the third part, target stimuli account for 25% of the items presented at a speed of 1000 milliseconds. In the final part, target stimuli account for 75% of the items presented at a speed of 2000 milliseconds. The task has been previously used by Trevisan [34] and showed reliability and evidence of validity, including increase in the performance with school progression, correlation with other executive functions instruments, and poor performance in children with ADHD in comparison with neurotypical children [34]. A 14″ touchscreen monitor connected to a notebook was used in this study. Four measurements were used: correct responses and reaction time for both the Go and No-Go items.

To evaluate cognitive flexibility and inhibition, the Five Digits Test (FDT) was used [35]. This allows the recognition of the progressive automatization of a task and the subject’s ability to switch between one rule and another. The test is divided into four parts with different difficulty levels. Each part is presented on a sheet with 60 boxes. Parts 1 and 2 assess automatic processes: in Part 1, the examinee reads Arabic numerals on a screen (1, 2, 3, 4, 5), and, in Part 2, counts asterisks (1–5 asterisks). In Part 3, the examinee should identify the number of digits (ignoring the digit reading). In Part 4, the task alternates between the rules given in Part 1 and Part 3: the participant counts the number of digits but must switch the rule and say what the digit is whenever there is a black border on the item. FDT has validity evidence for children from 6 years old up to adults, with analysis of factorial structure, correlations with other executive functions measures, and reliability (internal consistency and test–retest stability) [35]. All raw data were converted to *Z*-scores, and subsequently to percentiles, based on normative data standardized to the Brazilian population referenced in Sedó, De Paula, and Malloy-Diniz [35] and Miotto [36], according to each participant’s age. In this study, the two executive function measures for execution time were used: Inhibition (Part 3 minus Part 1) and Flexibility (Part 4 minus Part 2) [35].

### 2.3. Intervention Tools

#### 2.3.1. Experimental Group: Intervention in Executive Functions

An intervention program was developed for children in the second to fifth grades of primary school, based on the GMT technique. This is a metacognitive intervention that integrates psychoeducation, cognitive training, homework, and performance feedback, assimilating the content into the participants’ daily individual activities between sessions [19].

**Figure 1:**
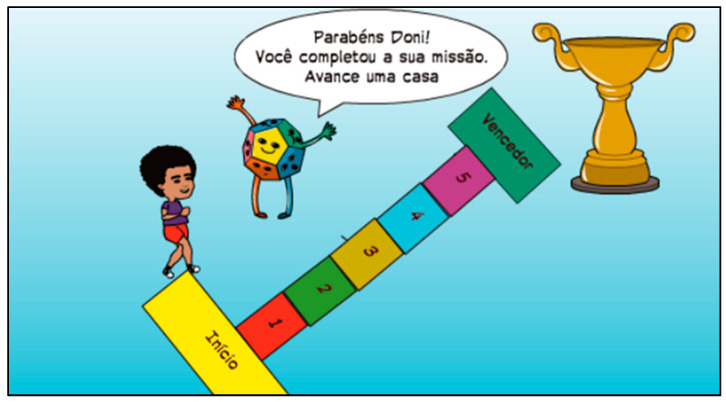
Example I of the “board game” psychoeducation tool (Executive functions: “Congratulations, Doni. You completed the mission. Move one space forward.”. Hopscotch: start; numbers refer to stages of the game; winner).

For this study, a comic story titled “The Adventures of Doni in the Mind-Connect Game” was created. This story, developed using the Pixton program, features the main character, a boy named Doni, as a model and narrator who encounters difficulties in achieving goals. Throughout the story, psychoeducational activities and the application of key GMT concepts are carried out, based on previous studies [19,20,22,25]. In the plot, Doni discovers a chest that serves as a gateway to the human board game, as illustrated in Figure 1. In this game, he encounters the “executive functions” team who seek help in their training.

**Figure 2:**
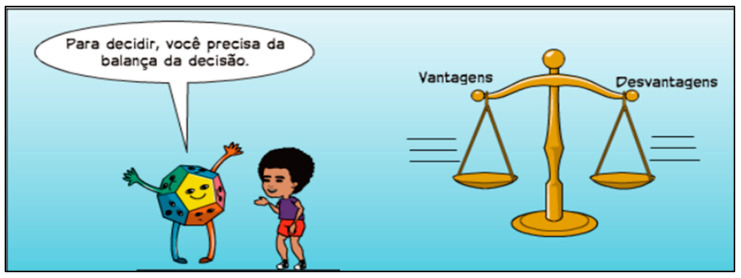
Example II of the psychoeducation tool (Executive functions: “To decide, you need the decision balance.” Balance: “Advantages” and “Disadvantages”).

**Figure 3:**
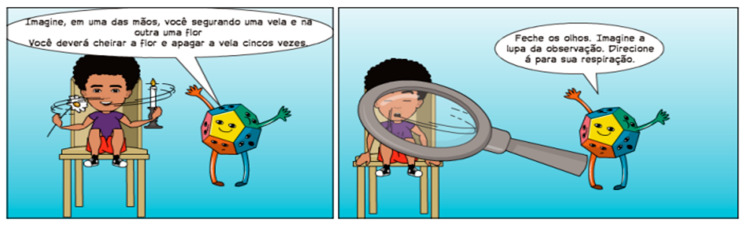
Example III of the psychoeducation tool (Executive functions, box 1: “Imagine, in one hand, you’re holding a candle, and in the other, a flower. You must smell the flower and blow out the candle five times.” Executive functions, box 2: “Close your eyes. Imagine the observation lens. Direct it towards your breath.”).

At each game space, a module of the GMT is introduced, and Doni is given a mission to be fulfilled in the real world. To accomplish the mission, Doni gains powerful tools, which are cognitive strategies, such as the “Observation Lens” representing self-monitoring, the “Stop Card” representing inhibitory control, the “Mind Agenda” representing working memory, the “Objective Card” representing goal setting, the “Decision Balance” representing decision-making, and the “Steps to the Goal” representing sub-goals, as depicted in Figure 2 and Figure 3. To apply the concepts in their daily activities during the week, the participants recorded the construction of their own stories in a comic format, following the model character.

The intervention introduces the key concepts of the GMT and comprises 6 modules, with a total of 38 activities, with each module corresponding to a key concept, namely: (1) attention, (2) autopilot, (3) working memory, (4) goals, (5) decision-making, and (6) planning [19]. In this study, module 1 sought to stimulate self-monitoring/attentional focus skills; module 2, inhibitory control skills; module 3, working memory skills; module 4, organization skills; module 5, cognitive flexibility skills; and module 6, planning skills. The experimental group received 16 intervention sessions, all conducted in groups with a duration of 1 h 30 min. Table 1 provides a summary description of the modules and the number of sessions and activities.

#### 2.3.2. Control Group

The active control group engaged in activities with themes related to citizenship. This choice was deliberate because citizenship-related topics are integral to fostering positive relationships among children, as well as between children and their environment. Importantly, the content chosen for the control group’s activities intentionally had minimal overlap with the executive function, the primary focus of the experimental group.

The active control group engaged in a series of activities spanning 16 sessions, each lasting 1 h and 30 min, which corresponded to the time of administration and the collective application of the GMT technique. The sessions covered the following sequence of discussion topics: Session 1—differences; Session 2—social exclusion; Session 3—non-violence; Session 4 and Session 5—coexistence; Session 6—friendship; Session 7—environment and preservation; Session 8—good manners; Session 9—solidarity; Session 10—children’s rights; Session 11—children’s duties; Session 12—responsibility; Session 13—rules for what?; Session 14—communication; Session 15—sharing knowledge; and Session 16—action and citizenship. The sessions were conducted using drawing, painting, storytelling, and dynamic activities to raise awareness among the participants regarding citizenship.

### 2.4. Procedure

The study was approved by the Institutional Ethics Committee of the Universidade Presbiteriana Mackenzie (CAAE: 82373617.3.0000.0084) and followed the ethical precepts for research with human subjects. Informed consent was obtained from all subjects involved in the study. Initially, the first author of this article met with all teachers from the second to the fifth grade who participated in the research. The concept of executive functions was explained, and descriptions related to executive functions were provided, using examples that could be observed in the classroom. Teachers were asked to indicate students in their classes who presented difficulties with executive functions, i.e., in working memory, inhibitory control, and/or cognitive flexibility. A total of 45 children were suggested.

Following this, the parents of the students indicated by the teachers were invited to a meeting. The parents of 43 students participated. The researcher explained the study to the attending parents, and they were invited to participate in the project along with their children. Everyone present agreed to participate in the project. They received the consent form, and then the IFERA-I and SDQ instruments described earlier. Two researchers assisted in completing the forms for illiterate parents. Subsequently, the teachers also completed the two scales for the students they indicated.

Assessment 1 (baseline) was conducted by two expert neuropsychologists. In order to minimize inter-examiner error, the examiners were properly trained in using the instruments, blinded to the study’s design, and randomly assessed children from both groups. The participants underwent a series of neuropsychological tests. An initial assessment lasted approximately 1 h 30 min. The following tests were administered in this sequence: WASI (to assess IQ), FDT (Executive functions), WISC-IV Digits (Working memory), and Go/No-Go Task (Inhibitory control).

After the initial assessment, the participants were randomly divided into two groups, the experimental group (EG) and the control group (CG). Then, each group was divided into three subgroups to match the session times with the schedules provided by the teachers for research activities. Accordingly, two experimental subgroups were in the morning and one in the afternoon, and two control groups were in the morning and one in the afternoon. The mean age of the EG was 108.7 months (SD = 10.08), and that of the CG was 111.06 months (SD = 11.97). The demographic profile of the two groups is shown in Table 2. There were no significant differences between the two groups in total IQ (*p* = 0.918), verbal IQ (*p* = 0.597), or performance IQ (*p* = 0.665).

All groups received 16 intervention sessions. The meetings took place at the school, in two rooms provided by the coordinators, lasting 1 h 30 min, twice a week, during the school period. The EG participants were present for 12 to 16 sessions (median of 14.5), while the CG participants were present for 13 to 16 sessions (median of 16). A 75% attendance rate was the minimum required for participation in this study, as we chose to respect the same minimum attendance frequency required by the school, considering that this program aims to be applied in a school context.

**Figure 4:**
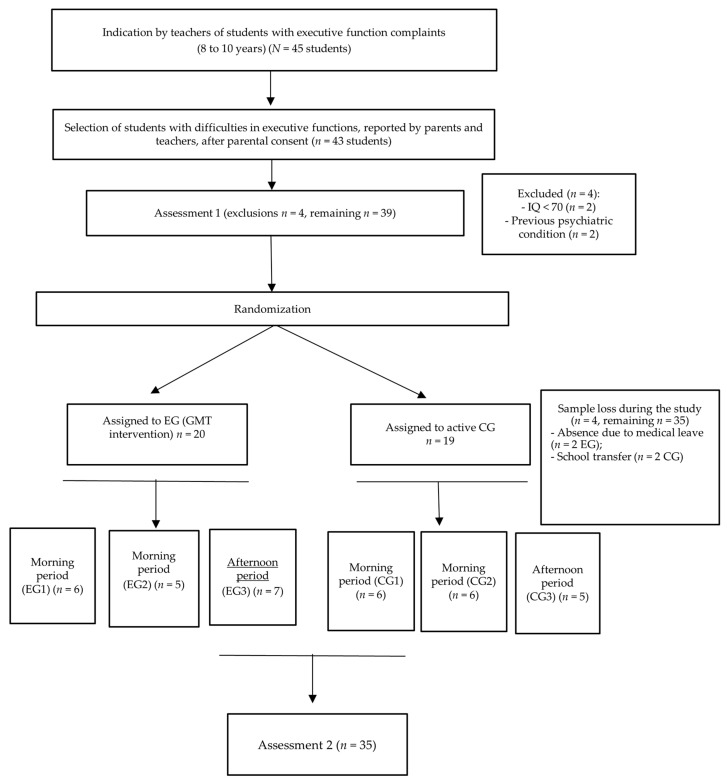
Study flowchart with the division of the study groups.

After the interventions, both groups, EG and CG, underwent a reassessment (Assessment 2) with the same instruments from the pre-test. The data collection was carried out by the same neuropsychologists of Assessment 1, who were unaware of the study design and the participant’s group allocation to avoid assessment biases. It should be emphasized that the parents and teachers were also blinded to the study design, meaning they were unaware of the groups to which the children were allocated (EG or CG). Figure 4 presents the flowchart of the study.

### 2.5. Analysis of the Results

Descriptive analyses of the investigated variables were conducted using measures of central tendency and dispersion, followed by the Shapiro–Wilk normality test. Even though there was no rejection of the null hypothesis in this test for most variables, due to the small sample size, two-way Wald-type statistic (WTS) was used to compare differences in primary outcomes for the factors of time and groups, using the R package nparLD 2.2 [37]. Relative treatment effect (RTE) was reported as a measure of effect size. RTE represents a probability that a randomly obtained observation, from a specific group, has a more significant value than that from the combined mean distribution [38]. RTE is a probability value between 0 and 1, with 0.5 signifying no effect and 0 and 1 signifying complete separation of the two groups (≥0.71 or ≤0.29: high effect; ≥0.64 or ≤0.36: medium effect; ≥0.56 or ≤0.44: low effect) [39]. *p* values were corrected for multiple comparisons using the false discovery rate (FDR) procedure, and PFDR < 0.05 were declared to be statistically significant. Python 3.6.5 and R version 3.6.1 were used to perform the analyses. No missing value treatment was used, adopting listwise deletion for each analysis.

## 3. Results

Table 3, Table 4 and Table 5 present the summary of the descriptive statistics obtained at both time points (pre-test and post-test) for both the CG and EG, along with the results of the two-way Wald-type statistic (WTS) (Time of assessment x Group), displaying the *p*-values for the main effect of the time of assessment, the *p*-value for the interaction between time and group, and the relative treatment effect of the interaction (RTE).

Table 3 specifically presents the summary of the results of the tests administered to the children. There was an effect of time on the Total IQ of the WASI, with both groups displaying increased performance. However, there was no significant interaction between them.

Concerning the Digits measure, assessing working memory, there was a marginally significant effect for the time of assessment, and a significant interaction between them was observed, with a low effect size, indicating a more considerable increase in the EG compared to the CG. In inhibition, measured by the FDT, there was a time effect, showing an increase in performance in both groups; however, there was no significant interaction between them, with a low effect size. Similarly, for the cognitive flexibility measure, also assessed by the FDT, there was a time effect with a marginally significant interaction and a moderate effect size, suggesting a greater gain in the EG compared to the CG.

Regarding the correct responses in the Go items of the Go/No-go test, there was no effect of time or significant interaction, displaying a low to medium effect size. While for the correct responses in the No-Go items, there was no time effect; however, there was a significant interaction between them was observed, with a low effect size, revealing a greater gain in the EG compared to the CG. In terms of time, for both the Go and No-Go items of the Go/No-go test, there was no time effect or significant interaction, displaying an insignificant effect size in the Go items but a low effect size in the No-Go items, indicating a more considerable decline in time for the EG.

Table 4 summarizes the results of the IFERA reported by parents and teachers. According to the table, there was a significant main effect of Assessment Time in all measures reported by teachers, indicating a decrease in difficulties in all areas, but not in the measures reported by parents. There was a significant interaction between Assessment Time and Group for four measures. According to the parental reports, there was a significant interaction in inhibitory control, with a low effect size. According to the teachers’ reports, there was a significant interaction in Delay Aversion (moderate effect size), Regulation (moderate effect size), and Total (high effect size). Additionally, in the teachers’ reports, there was a marginally significant interaction in inhibitory control (high effect size) and cognitive flexibility (moderate effect size). In all cases, the differences were in favor of the experimental group, suggesting a reduction in difficulties for this group.

Table 5 summarizes the results of the SDQ reported by parents and teachers. According to the table, there was no significant main effect of Assessment Time in any measure. There was only one significant interaction, in the Hyperactivity measure as reported by parents, with a low effect size, suggesting a greater reduction in difficulties in the EG compared to the CG. It should be highlighted that there was a marginally significant interaction for Relationship Problems as reported by parents, the only measure indicating a trend of greater reduction in difficulties in the CG than in the EG. This fact provides evidence that the control intervention applied to the CG was marginally effective, as the intervention focused on citizenship, relationships with people, and the environment, which may explain the improvement in this type of behavior in the CG.

## 4. Discussion

This study aimed to identify the gains from an executive function intervention based on the GMT approach in children aged 8 to 10, with complaints of executive function difficulties [19]. The intervention included mindfulness instructions, psychoeducation, self-instruction, and metacognition techniques. Evaluations were carried out through performance tests with the children and covered the fundamental executive function components (working memory, cognitive flexibility, and inhibition). Additionally, complementary behavioral measures were included based on reports from parents and teachers.

The results Indicated that children participating in the GMT training benefited in certain executive function measures, showing significant improvements in working memory and inhibitory control, and marginally significant gains in cognitive flexibility, as well as, to a lesser extent, in the reports provided by parents and teachers. In only one of the measures, the CG had a marginally greater gain than the EG, specifically in the measure of behavioral problems in the SDQ, possibly due to playful activities related to citizenship. It is important to highlight, as described in the Method section, that parents, teachers, and assessors were blind to the participants’ experimental conditions.

Regarding the total IQ, the gains in the experimental and control groups did not differ significantly and presented a negligible effect size. This result was expected for this study. Although higher-order executive functions, such as reasoning, are related to fluid intelligence according to some authors, such as Diamond [40], the intervention does not aim to directly promote changes in intelligence but rather uses this measure as a control [41].

In relation to working memory, assessed by the weighted score in the Digits subtest, the analyses revealed a significant interaction between group and assessment time, suggesting benefits from the GMT training. This increase in working memory might be due to the training with the “mind agenda” module, which included learning and application of metacognitive strategies in activities in and out of the sessions, suggesting that GMT, as observed in a previous study, could contribute to improved performance in working memory [26].

Regarding the FDT test, although there was no significant interaction, the marginally significant interaction, with a moderate effect size, for the flexibility measure, should be highlighted. This suggests an improvement in the performance of the EG in conducting the task. The EG exhibited faster performance in the alternation measure of this test, which aligns with previous studies involving interventions related to GMT [19,26].

In terms of inhibitory control, measured in the computerized Go/No-go task, the children in the EG achieved greater gains in the No-Go items, with a large effect size. This result indicates an improvement in inhibitory control, as in the task the child is expected to inhibit the impulse to press the button when presented with incongruent stimuli and to press correctly when presented with the target stimulus. Inhibitory control is one of the cornerstones of GMT, and a previous study also reported gains in this skill [26]. No statistical differences were found in the time measures.

Some important effects were observed in the instruments completed by the parents and teachers. In the IFERA-I completed by parents, there was a statistically significant gain in inhibitory control for the EG, with a moderate effect size, meaning the parents reported a decrease in the children’s difficulties in inhibitory control. In the IFERA-I completed by teachers, there was a significantly greater gain for the EG (i.e., a decrease in difficulties) in the measures of the total score, delay aversion (with a moderate effect size), and regulation (with a large effect size), along with a marginally significant interaction in inhibitory control and cognitive flexibility (moderate effect size). It should be highlighted that both parents and teachers were blind to the experimental condition, meaning they did not know to which group the children were assigned. These data underscore the importance of the findings and corroborates the performance test results, revealing gains in executive functions following the GMT. Additionally, the effects in the reports from the parents and teachers suggest that the gains from the intervention transferred to school and home settings. It is important to emphasize that in no measure did the CG have greater gains with moderate/large effect sizes or with significant or marginally significant differences. Therefore, the reports indicate that the children in the EG showed better executive functions not only in the structured tests applied, but also in behaviors at school and at home. This is consistent with the meta-analysis of Stamenova and Levine [25], where gains in everyday executive function activities were observed.

The gains observed in executive functions possibly explain the improvements noted in the participants’ behaviors, as reported by the parents and teachers using the SDQ. In this questionnaire, parents reported a significantly greater reduction in hyperactive behavior in children of the EG compared to the CG, with a large effect size. On the other hand, in the measure of relationship problems according to the parental reports, there was a marginally significant interaction, with a moderate effect size, indicating greater gains in the CG. This finding is particularly interesting and is likely due to the fact that the CG was an active group with interventions focused on citizenship-related themes, which might have contributed to reducing difficulties in terms of relationships within this group.

When comparing the effect sizes of traditional test measures in this study with a previous one that verified the effectiveness of GMT in a sample of adolescents [26], it is possible to note that both studies revealed a moderate effect size on cognitive flexibility, using traditional test measures immediately after the intervention. Also, both studies identified only small effect sizes on working memory following GMT intervention. However, unlike the study in adolescents, which identified a moderate effect size on inhibitory control following GMT intervention, the current study demonstrated a low effect size. When comparing the IFERI scale measures that assessed executive functions in the social environment, we also found higher size effects in this study, especially according to the teachers’ reports, with high effect sizes on inhibition and score total, and moderate effect sizes on cognitive flexibility, delay aversion, and state regulation. In the study with adolescents, only medium effect size for the active control group was observed [26]. This difference, with more evident results in the present study, can be due to the age of the participants and, also, to the number of sessions: 16 sessions in this study and only 8 sessions in the study with adolescents [26]. This result can suggest that a longer intervention is more able to promote gains.

In summary, the results indicate some evidence that the intervention was at least partially effective in developing executive functions in children. Considering the performance tests used to evaluate executive functions, there were significant effects in some measures across all three basic components of executive functions: working memory, inhibitory control, and cognitive flexibility (Digits, Go/No-Go, FDT). Additionally, changes were reported by the parents and teachers through the IFERA and the SDQ. The present study showed these significant results even when comparing the experimental group to an active control group, what is important because, in this type of comparison, effect sizes tend to be reduced [42].

The observed changes may be attributed to the activities conducted during the intervention. For instance, when working with the “Stop!” card tool, the intervention may have prompted children to reflect before making decisions, consequently reducing impulsive behavior. The “Decision Making Skill” module, utilizing the “Decision Balance” tool, may have fostered cognitive flexibility in children by encouraging reflection on the pros and cons of different choices. The use of the “Mind Agenda” may have contributed to the development of working memory and the application of mnemonic strategies by the children. It is worth noting that, by employing such activities ecologically, applying them to situations that genuinely form part of the children’s day-to-day lives, in a group context, a greater effect is expected, with more pronounced evidence of gains in executive functions, when compared to more structured interventions that are removed from the child’s real environment [6,39,40,41].

In this way, the study provided evidence for the feasibility and efficacy of the GMT intervention for children experiencing executive function difficulties, as reported by parents and teachers. However, it is a pilot study, and some limitations need to be highlighted. Primarily, the study sample was small, which might have introduced sample bias, since in a small sample, specific characteristics of participants may lead to differences between the groups, such as familial support or other cognitive skill variations not measured in this study. These possible confounding factors were not controlled in this research. Additionally, the results may not generalize well to a larger population, and the small sample size could contribute to the absence of significant effects in the Wald-type statistic in some measures.

Another limitation was the lack of a follow-up evaluation. Previous studies suggest that the effects of executive function training might be more evident in follow-up assessments rather than immediately after the intervention [19,26]. Therefore, it is suggested that future studies address these limitations by incorporating a larger and more diverse sample, controlling other variables, as well as including follow-up assessments.

## 5. Conclusions

Despite the limitations, this pilot study allowed for the preliminary development and investigation of an intervention proposal aimed at enhancing working memory, inhibitory control, and flexibility in children. The results, while modest, seem to indicate a promising direction for further research in this field. It is important to highlight that, although the GMT training was administered by a researcher in this study, it was developed to be carried out by teachers in a classroom setting, meaning it is entirely suitable for this context and can be used as a naturalistic school intervention [6]. Future research should test the program’s efficacy when implemented by teachers in the classroom with bigger samples, and analyze its long-term effects on cognitive abilities, behavior, and academic performance.

The use of interventions integrated into the school environment has been highlighted in the literature due to enabling greater transfer to other contexts compared to more specific and structured executive function training [43,44]. In addition to the greater transfer to untrained skills, implementing interventions in a school setting by teachers has the potential to reach more children, as all students can benefit from the activities, unlike interventions in clinical contexts that are limited to a relatively small portion of the child population [42]. Furthermore, training teachers to promote executive functions is highly generative, as a teacher can conduct the intervention with multiple classes throughout their professional practice.

However, it should be considered that the application by teachers poses some challenges. For instance, teacher training is necessary for the intervention implementation, and the classroom environment can be difficult, with a larger number of students and potentially greater diversity in terms of prior knowledge and cognitive and academic abilities. Additionally, the way teachers conduct the activities is a crucial factor that can determine the gains achieved by students [43]. Future research is required to examine the feasibility and potential effects in studies where teachers directly implement the intervention with students, as well as long-term effects and possible impacts on academic achievement. Despite these challenges and the need for further research, previous studies have revealed the efficacy of interventions conducted directly by teachers in the school context [43,44]. Therefore, the use of ecological interventions, especially those administered by teachers, can be a low-cost, effective, and far-reaching solution for promoting executive functions in children.

## Figures and Tables

**Table 1:** Summary of the six intervention modules.

Module	Structure	Description
**(1) Attentional skill**	Number of sessions	4
	Number of activities	12
	Tool	Observation lens
	Objective of the tool	Promoting self-monitoring
	Description of psychoeducation activities	Transmission of the overall concept of executive functions to children; promoting awareness of errors due to lack of attention and the consequences of these errors
	Description of cognitive training activities	Formulation of goals and intervention strategies in attentional skills; self-monitoring training; mindfulness practice to stimulate sustained and selective attention
	Description of homework tasks	Self-monitoring practice according to the list of goals formulated in the first session; mindfulness
	Bridges between sessions and review of homework tasks.	Reviewing the concepts learned in the previous session by asking questions to relate to the current session; checking whether the child completed the homework, and discussing successes and difficulties
**(2) Inhibitory control skill**	Number of sessions	3
	Number of activities	8
	Tool	“Stop!” card
	Objective of the tool	Promoting inhibitory control
	Description of psychoeducation activities	Introduction to the concept of being on “autopilot”; developing the ability to control impulses in automated situations; application of metacognitive strategies to develop self-control
	Description of cognitive training activities	Practice of “Stop the autopilot!” aimed at stimulating attention, self-monitoring, and inhibitory control; a “stop” game for inhibitory control training, applying self-monitoring; reading colored numbers to stimulate inhibitory control; mindfulness practice
	Description of homework tasks	Practice stopping the “autopilot” in predetermined situations in the intervention planning; mindfulness
	Bridges between sessions and review of homework tasks.	Reviewing the concepts learned in the previous session by asking questions to relate to the current session; checking whether the child completed the homework, and discussing successes and difficulties
**(3) Working memory skill**	Number of sessions	2
	Number of activities	5
	Tool	Mind agenda
	Objective of the tool	Promoting working memory
	Description of psychoeducation activities	Introducing the concept of working memory; applying mnemonic strategies
	Description of cognitive training activities	Stimulating visual and auditory working memory through games with standardized sounds; using the metacognitive strategy of stopping and consulting the mind’s agenda at timed intervals; mindfulness
	Description of homework tasks	Daily practice of the stop-and-consult strategies at timed intervals in the mind agenda; mindfulness
	Bridges between sessions and review of homework tasks.	Reviewing the concepts learned in the previous session; assessing the application of these concepts in the week before the session
**(4) Goal setting/prioritization skill**	Number of sessions	2
	Number of activities	6
	Tool	Objective card
	Objective of the tool	Promoting goal representation
	Description of psychoeducation activities	Introducing the concept of goal selection; presenting the metacognitive strategy (stop, breathe, and ask yourself “What is my goal?”)
	Description of cognitive training activities	Activities involving pencil and paper; categorizing cards by color using the metacognitive strategy; mindfulness
	Description of homework tasks	Practicing “goal setting” using the self-instruction strategy; mindfulness
	Bridges between sessions and review of homework tasks.	Reviewing the concepts learned in the previous session; checking whether the planned goals were achieved
**(5) Decision-making skill**	Number of sessions	2
	Number of activities	4
	Tool	Decision balance
	Objective of the tool	Promoting decision-making
	Description of psychoeducation activities	Introducing the concept of decision-making and its implications in automated mechanisms
	Description of cognitive training activities	Activity “decide the end of the story” to train decision-making; using the “decision balance” technique to deal with advantages and disadvantages; mindfulness
	Description of homework tasks	Implementing the “decision balance” technique in the child’s daily activities to promote generalization; mindfulness
	Bridges between sessions and review of homework tasks.	Reviewing the concepts learned in the previous session about decision-making; checking the application of these concepts in the week before the session
**(6) Planning/organization skill**	Number of sessions	3
	Number of activities	4
	Tool	Steps to the goal
	Objective of the tool	Promoting the construction of sub-goals
	Description of psychoeducation activities	Introducing the concept of breaking down a goal into sub-goals; presenting metacognitive strategies
	Description of cognitive training activities	Activity “organizing the backpack” to stimulate planning/organization; dividing complex tasks into smaller ones; self-assessment of performance; mindfulness
	Description of homework tasks	Implementing the learned strategies in everyday activities
	Bridges between sessions and review of homework tasks.	Reviewing the main concepts learned and linking them with the homework; assessing the difficulties or successes

**Table 2:** Demographic profile of participants.

	Education	N	Sex	Age	Race
**Experimental Group**	3th grade	11	6 M; 5 F	08	09 White; 02 Black
	4th grade	02	1 M; 1 F	09	02 White
	5th grade	05	3 M; 2 F	10	01 Black; 04 White
**Control** **Group**	3th grade	10	6 M; 4 F	08	10 White
	4th grade	03	2 M; 1 F	10	01 Black; 02 White
	5th grade	04	2 M; 2 F	10	02 Black; 02 White

Note: F = feminine; M = masculine.

**Table 3:** Descriptive statistics and two-way Wald-type statistic (WTS) results in the assessment tests.

Variable	Group	Mean (SD) Pre-Test	Mean (SD) Post-Test	Time *p*-Value	Time*Group *p*-Value	Relative Treatment Effect (RTE)
IQ total	E	89.94 (10.95)	98.06 (14.19)	<0.001	0.736	0.60
	C	89.71 (10.59)	97.47 (14.63)			
Digits	E	7.17 (2.50)	8.94 (2.13)	0.053	0.044	0.61
	C	7.94 (2.13)	7.65 (2.50)			
FDT Inhibition	E	45.28 (27.84)	63.61 (35.39)	0.021	0.209	0.58
	C	55.29 (29.45)	61.18 (33.38)			
FDT Flexibility	E	43.89 (32.43)	70.28 (29.28)	0.008	0.073	0.64
	C	51.06 (27.74)	56.47 (29.36)			
Go/No-go Go items score	E	0.91 (0.03)	0.92 (0.05)	0.718	0.363	0.58
	C	0.85 (0.17)	0.82 (0.15)			
Go/No-go No-go items score	E	0.87 (0.08)	0.93 (0.04)	0.051	0.002	0.61
	C	0.91 (0.06)	0.89 (0.11)			
Go/No-go Go items reaction time	E	0.59 (0.06)	0.58 (0.06)	0.432	0.447	0.47
	C	0.62 (0.09)	0.61 (0.09)			
Go/No-go No-Go items reaction time	E	0.47 (0.07)	0.44 (0.09)	0.978	0.348	0.44
	C	0.47 (0.10)	0.59 (0.37)			

**Table 4:** Descriptive statistics and two-way Wald-type statistic (WTS) results in the IFERA reported by parents and teachers.

Variable	Group	Mean (SD) Pre-Test	Mean (SD) Post-Test	Time *p*-Value	Time*Group *p*-Value	Relative Treatment Effect (RTE)
**IFERA Parents**						
Inhibitory control	E	3.22 (0.83)	2.83 (0.79)	0.233	0.05	0.39
	C	3.12 (0.60)	3.24 (0.81)			
Working memory	E	3.12 (0.95)	2.81 (0.94)	0.165	0.252	0.43
	C	3.04 (0.97)	3.05 (1.10)			
Cognitive flexibility	E	3.00 (0.87)	2.71 (0.67)	0.107	0.297	0.42
	C	2.92 (0.71)	2.90 (0.90)			
Delay aversion	E	3.27 (0.96)	2.98 (0.87)	0.555	0.611	0.45
	C	3.17 (0.73)	3.16 (0.91)			
State regulation	E	3.53 (0.89)	3.23 (0.87)	0.064	0.434	0.41
	C	3.58 (0.58)	3.42 (0.90)			
Total	E	3.24 (0.71)	2.93 (0.75)	0.084	0.211	0.41
	C	3.19 (0.45)	3.17 (0.67)			
**IFERA Teachers**						
Inhibitory control	E	3.54 (0.72)	2.71 (0.60)	<0.001	0.083	0.28
	C	3.74 (0.78)	3.63 (1.50)			
Working memory	E	3.73 (0.85)	2.77 (0.80)	<0.001	0.822	0.31
	C	3.93 (0.84)	3.13 (0.74)			
Cognitive flexibility	E	3.65 (0.50)	2.88 (0.70)	<0.001	0.082	0.31
	C	3.65 (0.55)	3.37 (0.80)			
Delay aversion	E	3.24 (0.87)	2.18 (0.91)	<0.001	0.041	0.30
	C	3.25 (0.60)	2.97 (1.11)			
State regulation	E	3.76 (0.72)	2.88 (0.98)	<0.001	0.05	0.34
	C	3.57 (0.65)	3.41 (0.82)			
Total	E	3.57 (0.51)	2.69 (0.62)	<0.001	0.029	0.25
	C	3.63 (0.48)	3.31 (0.71)			

**Table 5:** Descriptive statistics and two-way Wald-type statistic (WTS) results in the SDQ reported by parents and teachers.

Variable	Group	Mean (SD) Pre-Test	Mean (SD) Post-Test	Time *p*-Value	Time*Group *p*-Value	Relative Treatment Effect (RTE)
**SDQ Parents**						
Total	E	25.59 (3.87)	23.76 (5.08)	0.397	0.609	0.44
	C	24.87 (9.49)	25.87 (11.11)			
Emotional symptoms	E	3.94 (1.56)	3.35 (1.69)	0.341	0.294	0.42
	C	4.45 (2.35)	4.55 (3.06)			
Conduct problems	E	3.47 (1.01)	3.41 (1.42)	0.781	0.429	0.48
	C	3.53 (1.77)	4.12 (2.62)			
Hyperactivity	E	5.06 (1.81)	3.94 (1.48)	0.676	0.013	0.39
	C	4.60 (2.23)	5.63 (2.43)			
Relationship problems	E	4.71 (0.92)	5.23 (1.71)	0.514	0.058	0.51
	C	5.82 (2.25)	4.93 (2.28)			
Prosocial behavior	E	8.41 (2.00)	7.82 (1.78)	0.458	0.264	0.48
	C	7.51 (2.28)	7.66 (2.24)			
**SDQ Teachers**						
Total	E	20.61 (4.54)	19.89 (3.48)	0.971	0.631	0.51
	C	18.73 (4.71)	20.07 (4.38)			
Emotional symptoms	E	3.61 (2.59)	3.00 (1.68)	0.809	0.710	0.49
	C	3.33 (2.87)	3.46 (2.75)			
Conduct problems	E	3.72 (2.05)	3.17 (1.80)	0.911	0.075	0.44
	C	3.53 (1.73)	4.07 (2.12)			
Hyperactivity	E	5.22 (1.93)	4.94 (1.80)	0.808	0.292	0.48
	C	4.73 (1.87)	5.13 (1.30)			
Relationship problems	E	3.89 (2.17)	4.39 (1.38)	0.139	0.368	0.58
	C	3.76 (1.64)	3.88 (2.18)			
Prosocial behavior	E	7.06 (1.95)	7.89 (2.45)	0.132	0.174	0.61
	C	6.13 (3.54)	6.60 (2.64)			

## Data Availability

Data are available on request. The data are not publicly available due to [General Personal Data Protection Law, which is a local law, for scientific purposes data can be requested from the main author].
